# Anatomy and Ultrastructure of Galls Induced by *Neuroterus quercusbaccarum* (Hymenoptera: Cynipidae) on Oak Leaves (*Quercus robur*)

**DOI:** 10.3390/insects12100850

**Published:** 2021-09-22

**Authors:** Leszek Stanisław Jankiewicz, Marzenna Guzicka, Agnieszka Marasek-Ciolakowska

**Affiliations:** 1 The National Institute of Horticultural Research, ul. Konstytucji 3 Maja 1/3, 96-100 Skierniewice, Poland; 2 Institute of Dendrology, Polish Academy of Sciences, Parkowa 5, 62-035 Kórnik, Poland; guzicka@man.poznan.pl

**Keywords:** Cynipidae, plant–insect interactions, spangle gall, *Quercus robur*

## Abstract

**Simple Summary:**

Galls induced by insects are commonly found on oak leaves in temperate climates. Their induction and development are, however, still only partially known. Our aim was to present a detailed description of their anatomy and fine structure. Using scanning electron microscopy (SEM), we observed the wax cover of a gall and two types of openings on its surface: one similar to degenerated stomata and the other one that we called “large openings on epidermal protuberance”. Nutritive tissue underwent marked changes with time: in a young gall, the cellular walls were not visible, and a distinct cellular structure appeared only later. In the mound of the gall, there was a distinct subepidermal layer of cells containing dense cytoplasm. Tannins occurred in vacuoles or in cell walls in granular form or in compact formations. The leaf subtending the gall showed additional cell divisions and strong lignification of cell walls in the tissue surrounding the peduncle of the gall.

**Abstract:**

The structure and ultrastructure of two developmental stages of the spangle gall induced by *Neuroterus quercusbaccarum* (Hymenoptera, Cynipidae) were investigated using light microscopy (LM), fluorescence microscopy (FM), and transmission (TEM) and scanning (SEM) electron microscopy. The general design of the gall structure was typical of Cynipidae, but some structural features distinguished the spangle gall. Previously undescribed, characteristic multicellular epidermal protuberances with large openings were observed in autumn on the surface of galls. These may facilitate the gas exchange between the atmosphere and the inside of the gall, thus assisting larval respiration. The larval chamber is surrounded by both a sclerenchymatous capsule and numerous cells containing calcium oxalate crystals that may both serve as protective barriers. In young galls, the nutritive tissue is a wall-less protoplasmic mass, potentially easily accessible to young larvae with delicate mandibles. Cell walls only develop at a later stage. The nutritive tissue was found to be rich in proteins and lipids, but starch grains were not observed. Cellular topology suggests that spangle galls grow by anticlinal division of marginal epidermal cells and periclinal division of subepidermal cells. Cellular proliferation (hyperplasia) also occurs in the leaf tissue near the connection with the gall peduncle, which eventually lignifies.

## 1. Introduction

The discussion on the nature of plant galls has lasted for more than 100 years [1], and we still know very little about how this kind of neoformation is induced and developed [2,3,4,5,6]. Investigations of gall anatomy and development are pivotal because they are likely to disclose important clues about still poorly known mechanisms of plant morphogenesis [3,7,8].

Individual host plants are often attacked by different wasp species that manipulate plant morphogenesis, inducing the development of species-specific gall phenotypes. The initiation and development of galls are controlled by the insect to create a novel pseudo-organ for its own purposes, entirely comprising plant tissue [9,10]. Cynipid galls are examples of induced plant development where an inducer controls plant-cell differentiation [11]. Structurally complicated galls protect larvae and provide them with nutrients [10,12]. Nutrients such as lipids, proteins, and sugars are accumulated in cells of nutritive tissue lining the larval chambers. Such cells, packed with nutrients, are not found anywhere else in the host plant [13].

Signalling between wasp and host plant is still unknown. The most widely accepted hypothesis explaining gall formation is through their hormonal activity [14,15,16,17,18], though studies so far have not been able to explain the complexity of wasp galls only by this method [19]. Some authors postulated also that the fluid excreted by the female during the deposition of eggs into the plant tissue [10] or chemical secretions covering the egg [20]) are involved in gall initiation. Hypotheses were also proposed according to which the mechanism of induction is related to symbiotic viral partners [21,22] or bacterial or fungal symbionts [10]. We proposed a novel interpretation for the mechanism of gall induction [23]: the species-specific response induced in the host plant by cynipid wasps is so complex as to suggest gene transfer from the insect to the plant. The genetic transformation hypothesis was postulated by Gätjens-Boniche [24] according to which gall formation is mediated by the insertion of exogenous genetic elements into the genome of plant gall cells through some endosymbiotic bacteria originating from the insect. However, because the gall stops growing and degenerates if the larva inside it dies, gall development appears to require a continuous flow of signals from the growing larva.

Spangle gall is one of the most common galls occurring on oak trees in Poland [25]. Cynipid wasp *Neuroterus quercusbaccarum* has both agamic and sexual generations (heterogony) that cause morphologically different galls to occur on oak trees. In April, from overwintered spangle galls asexual wasps (two different types of females—androphores, which produce haploid eggs that give rise only to males, and gynephores, which produce diploid eggs that give rise only to sexual females) emerge and induce the formation of currant galls, in which the sexual generation develops [20,26]. The currant galls (which we do not consider in this paper) are spherical, and in contrast to common gall their development is rapid [26]. Currant galls are not restricted to leaves and may appear on male oak inflorescences, bracts, or young stems [27]. In June, male and female (here also there are two types of females) emerge from currant galls, and after mating, fertilized eggs are laid in the abaxial epidermis of oak leaves, inducing the formation of flat, circular, 4–6 mm in diameter spangle gall is formed. Spangle galls usually appear in groups, even up to 120 galls per leaf. These complete development in October, detach before leaf abscission, fall to the ground, and are covered by leaf litter, which protects them from drying out and from frosts [26]. Inside, the larva matures and pupates [26]. The development of spangle galls takes more than four months [26]. It is important to note that the stage of leaf-blade expansion, and, thus, the state of the leaf cells, is different in April when unfertilized eggs are deposited, and in June when fertilized eggs are deposited [27].

The development of *N. quercusbaccarum* galls on pedunculate oak leaves has a negative impact on the host plant, including altering the cell membrane integrity, disturbing photosynthesis, and reducing antioxidant potential [28].

The spangle gall is a type of nutritive gall composed of three layers: the nutritional, parenchymal, and epidermal [29]. Its structure was partly described by Hough [27]; see also Meyer and Maresquelle [3], and Kovácsné Koncz et al. [30]. The aim of this paper is to expand the description of the spangle gall of *N. quercusbaccarum* with the use of more diverse microscopic methods (LM, FM, TEM, SEM). Some histochemical tests were also conducted to reveal the accumulation of food reserves in the different tissue types of the gall.

## 2. Material and Methods

### 2.1. Gall Collection

**Figure 1:**
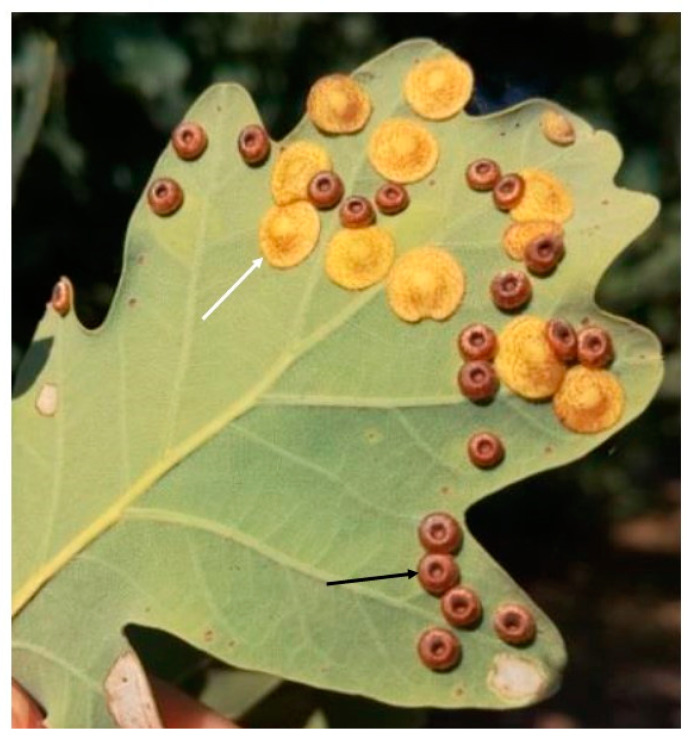
Galls of two species of Cynipidae on abaxial side of the oak leaf: *Neuroterus quercusbaccarum*, spangle gall (white arrow); *N. numismalis* (black arrow). Picture at mid-August.

The galls produced by the same-sex generation of *N. quercusbaccarum* were mainly collected in late July (“young gall”), when the galls were about half of their late autumn size, and in early October (“autumn gall”). Some observations were made on galls collected at other dates. Fallen galls and the larvae inside them pass the final stages of development during the cold part of the year under fallen leaves. The imago of the first generation is ready in early spring and produces ball-shaped galls [3,25,26]. The oak trees from which the leaves with galls were collected were about 5–25 years old and were growing in a mixed pine–oak forest, planted on medium-fertility soil near Skierniewice (central Poland). Normal oak leaves without galls collected at the same time served as the control. Spangle galls sometimes occurred together with galls of the allied species *N. numismalis* (Figure 1).

### 2.2. Cytochemical Investigations in Light (LM) and Fluorescence (FM) Microscopy

Parts of leaves with and without galls, and the galls themselves were prepared free hand in sections using a razor blade and observed unstained in LM (Eclipse 80i, Nikon, Tokyo, Japan) equipped with a polarization facility to detect starch, CaOx crystals, and lignins, or stained with Sudan III for lipid detection (positive results, orange) or HNO_3_ for protein detection. according to Gerlach [31] and Ruzin [32]. For histological study with LM, the galls with oak leaves were also fixed in a chromic acid–acetic acid–formalin (CrAF) mixture, dehydrated in a graded series of ethanol, and embedded in paraffin according to the method reported by Ruzin [32]. Cross-sections of 10 μm thickness were cut with a rotary microtome (Leica, Wetzelar, Germany), and stained with safranin and fast green following Gerlach [31]. Staining results are the purplish red of the lignified cell walls, nucleic acids, and green cytoplasm in the live cells. The material was analysed with a Nikon Eclipse 80i light microscope with NIS-Elements BR ver. 4.00 imaging software (Nikon Instruments Inc., Tokyo, Japan) for photo-documentation. At least 3–4 samples from different trees were taken for each investigation (trees that looked similar were chosen). Endogenous sources of fluorescence [33,34] in the live gall tissue were registered with confocal microscope Leica SP5 (Leica Microsystems, Wetzlar, Germany). Free-hand sections were used without staining for the spectral imaging of autofluorescence in the galls and leaves using different excitation [35]: for chlorophyll, 488 and 561 nm (emission at 500–569, 570–700 nm); for lignin UV, 355 and 458 nm light (emission at 465–700 nm); for tannin, 355 and 488 nm (emissions over the range of 500–650 nm).

### 2.3. Scanning Electron Microscopy (SEM)

For SEM, typical leaves and galls on a leaf surface were fixed with a chromic acid/acetic acid/formalin (CrAF) mixture/fixative, dehydrated in ethanol, desiccated with critical point drying CO_2_, and sputter-coated with gold [36]. The micromorphology of the gall surface, the oak-leaf surface, and the internal structure of galls were analysed using scanning electron microscope JEOL JSM 6390LV in Mossakowski Medical Research Centre, Polish Academy of Sciences in Warsaw.

### 2.4. Transmission Electron Microscopy (TEM)

For TEM, fragments of plant material (2 × 2 mm) were fixed in 2.5% glutaraldehyde for 12 h at 4 °C in 0.1M cacodylate buffer (pH 7.4) [6]. Then, they were rinsed several times in 0.1 M cacodylate buffer for 12 h. Afterwards, the material was treated with a 1% aqueous solution of osmium tetroxide for 4 h. After dehydration in ethanol and propylene oxide [37], the material was embedded in a mixture of epoxide resin [38] and cut using ultramicrotome LKB for semithin sections of 2 µm and ultrathin sections (about 80 nm). The ultrathin sections were then contrasted with uranyl acetate and lead citrate according to Reynolds [39]. Ultrathin sections were observed with transmission electron microscope JEOL JEM-1200EX in Mossakowski Medical Research Centre, Polish Academy of Sciences in Warsaw.

## 3. Results

### 3.1. Young Gall Collected in July—SEM Observations

**Figure 2:**
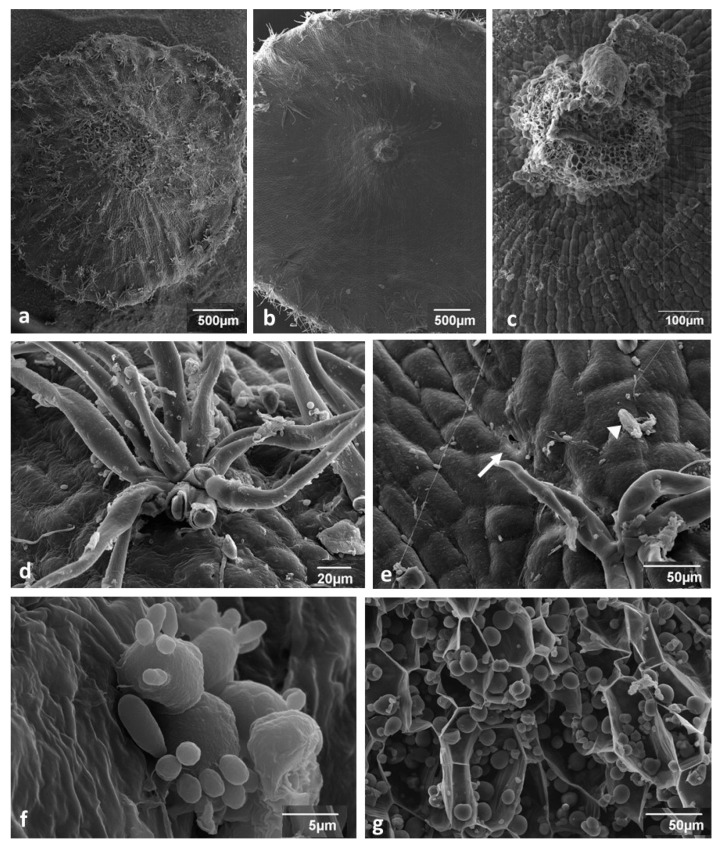
Young spangle gall collected at the end of July (SEM images). (**a**) Outer side with numerous tufted (stellate) hairs. (**b**) Inner side; in the centre is a broken stalk (peduncle) that attaches the gall to the abaxial side of the leaf. (**c**) Detail of a broken stalk showing radial files of gall epidermal cells. (**d**) Cluster of tufted hairs. Broken ends show extremely thick walls almost completely filling the cell lumen. (**e**) Small stomata-like opening (an arrow) on the outer surface of a gall. A spore of an unknown organism (an arrowhead) and a silk thread left by an insect or arachnid are also visible. (**f**) Yeastlike organism on abaxial surface of a gall. (**g**) Thin-walled gall parenchyma cells with abundant starch grains.

The outer side of the gall (Figure 2a) was covered with clusters of hairs (tufted or stellate hairs) that were densely crowded, especially at the central bulge (mound) (Figure 2a). The inner side (stalked surface; Figure 2b) was almost devoid of tufted hairs, and only some were found near the margins. The tufted hairs (Figure 2d,e) were 100–300 µm long and 12–20 µm thick at the base. They were sometimes curved or twisted (Figure 2d,e). Their number in one cluster was in the range of 7–20. An examination of broken ends showed that the hairs were not empty (Figure 2d). In the centre of the inner surface of the gall, there was the stalk (peduncle) attaching the gall to the abaxial leaf surface (Figure 2b,c). The stalk vessels were narrow and had helical thickenings, typical of the primary xylem.

**Figure 3:**
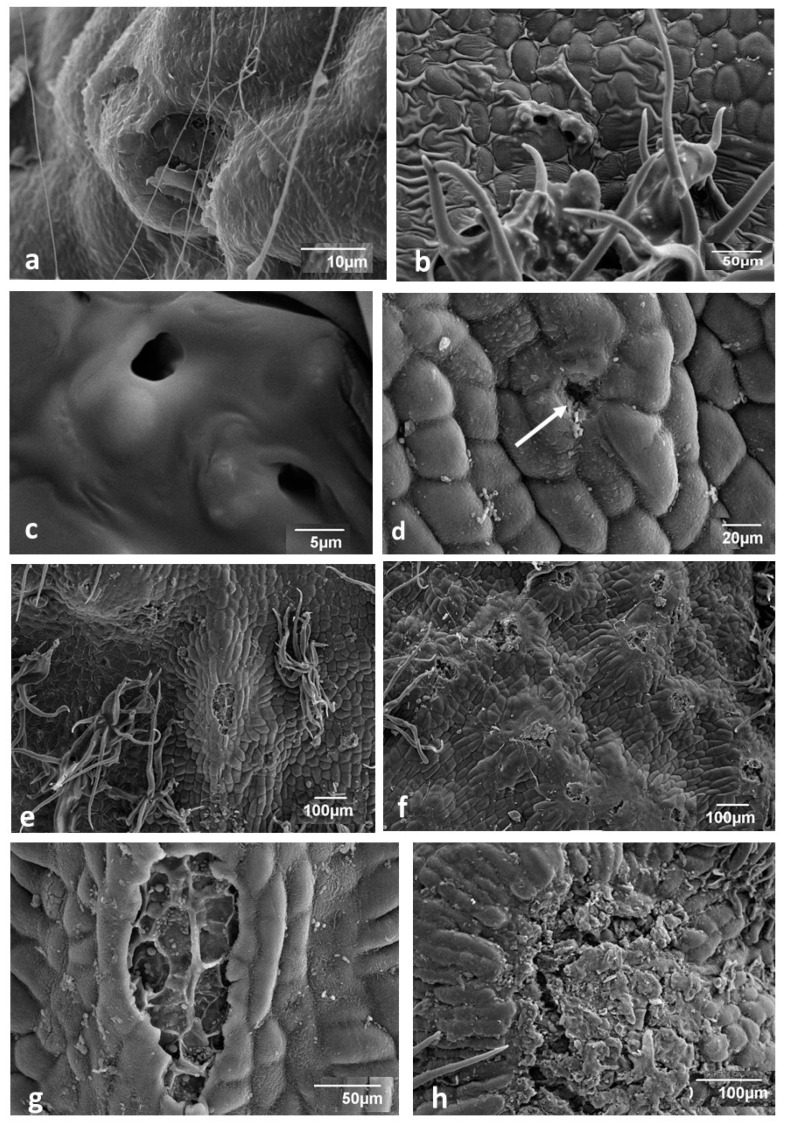
SEM of a gall collected in the middle of August. (**a**) Epidermal cells covered with a layer of wax (high magnification). (**b**) Wax layer was locally thicker, especially at junctions between cells. (**c**) Even when the cover of wax was thick, there were openings inside it. (**d**) Stomata-like openings dispersed on the outer side of the gall (compare with Figure 2e). (**e**) Large openings on epidermal protuberance. (**f**) Group of large openings. (**g**) Large opening on epidermal protuberance at a higher magnification, showing numerous microorganisms. (**h**) Breaks in subepidermal tissue may facilitate tissue aeration.

Epidermal cells on both sides of the gall were arranged in radial rows converging toward the centre (Figure 2c). Small openings were scattered on the gall surface. Some looked like normal stomata (Figure 2e) but others looked like non-functional, permanently open stomata (Figure 3d). A diversity of organisms and traces of their activity inhabited the surface of the gall, for instance, silk threads produced by arachnids or insects, unknown spores (arrowhead), bacteria (Figure 2e), yeastlike organisms (Figure 2f), and the hyphae of phyllospheric fungi (not shown). The young gall parenchyma (Figure 2g) was composed of thin walled densely-packed cells containing abundant starch grains.

### 3.2. Changes in Gall Surface during Development—SEM Observations

In August, both sides of the gall were covered with wax (Figure 3a). The epicuticular wax film was thicker in some places, especially at junctions between epidermal cells (Figure 3b), or it was uniformly thick but in this case, the openings were present (Figure 3c). At this time, openings similar to the degenerated stomata occurred on the outer side of the gall (Figure 2e and Figure 3d). In older galls in autumn, besides these small openings, there were also large ones with irregular edges, which were located on the top of multicellular epidermal protuberances (Figure 3e–g). They occurred on the surface of the gall singly or in groups (Figure 3f,g). Figure 3g shows that these large openings may serve as a shelter for microorganisms or even as a gateway to internal tissue. These large openings on epidermal protuberance may facilitate the exchange of gases between the atmosphere and the gall’s inside, thus assisting larval respiration. Occasional breaks in superficial gall tissue were also found (Figure 3h).

### 3.3. Changes Accompanying Gall Maturation (FM, LM—Polarized Light)

**Figure 4:**
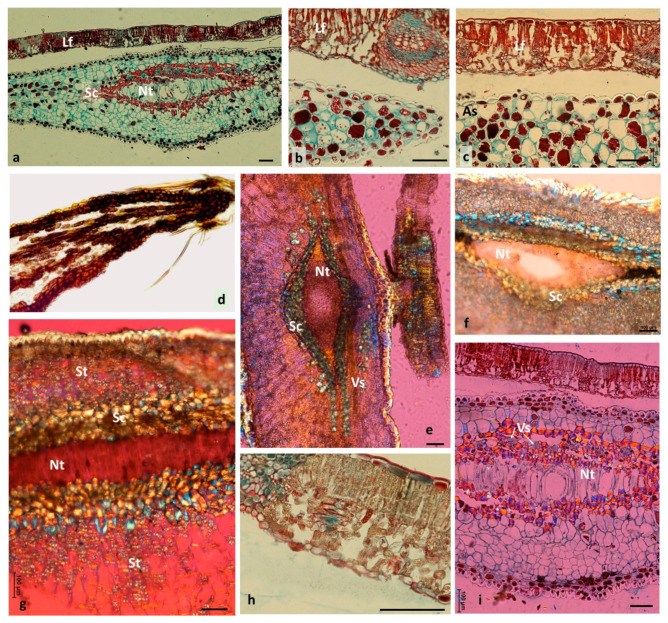
Spangle gall and its subtending leaf at different stages of development (LM, fast green and safranine, polarized light). (**a**) Cross-section near the middle of a gall. In the centre, the sclerenchymatous capsule (Sc) protecting the larval chamber, Nt, nutritive tissue; Lf, subtending leaf. Many cells show tannin deposits in the vacuole (brown). (**b**) Marginal part of a gall. (**c**) Cross-section through the inner part of a gall. As, large intercellular spaces looking as “breaks” in the gall subepidermal layer. Structure of the subtending leaf (Lf) markedly changed and heavily lignified. (**d**) Section of the gall taken in late July (not stained), irregular distribution of tannins. (**e**) Free-hand section taken in late July, not stained. Nutritive tissue shows granular structure. Cell walls not visible. (**f**) Another example of nutritive tissue without visible cell walls. Thick layer of CaOx idioblasts and sclereids above and below the larval chamber. (**g**) Cellular structure in the nutritive tissue already well-visible (mid-August). (**h**) Leaf blade beyond the influence of the gall; compare with degenerated leaf structure in (**c**). (**i**) Central part of a gall when nutritive tissue is already fully formed (late August). Bars represent 100 µm.

In mid-August, the most conspicuous trait of the gall is the larger size of its cells (Figure 4a–c) compared with the cells of the subtending leaf. Cells along the edge of the gall disc (Figure 4b) were relatively smaller than the other cells of the gall, and their cytoplasm appeared to be denser. Intercellular spaces in the gall parenchyma were mostly schizogenous and generally small; however, in the upper subepidermal layer (Figure 4c), there were larger spaces.

Epidermal cells on both sides of the gall had relatively thick external walls and were birefringent in polarized light, similarly to the hairs (a symptom of lignification; Figure 4e,g).

Tannin-filled cells were abundant in both the epidermis and the inner parenchyma (Figure 4a,d). Tannin cells in the parenchyma were scattered among tannin-free cells (Figure 4b,c). In a typical oak leaf, cells of the adaxial epidermis frequently contained abundant deposits of tannins (Figure 4c,h).

**Figure 5:**
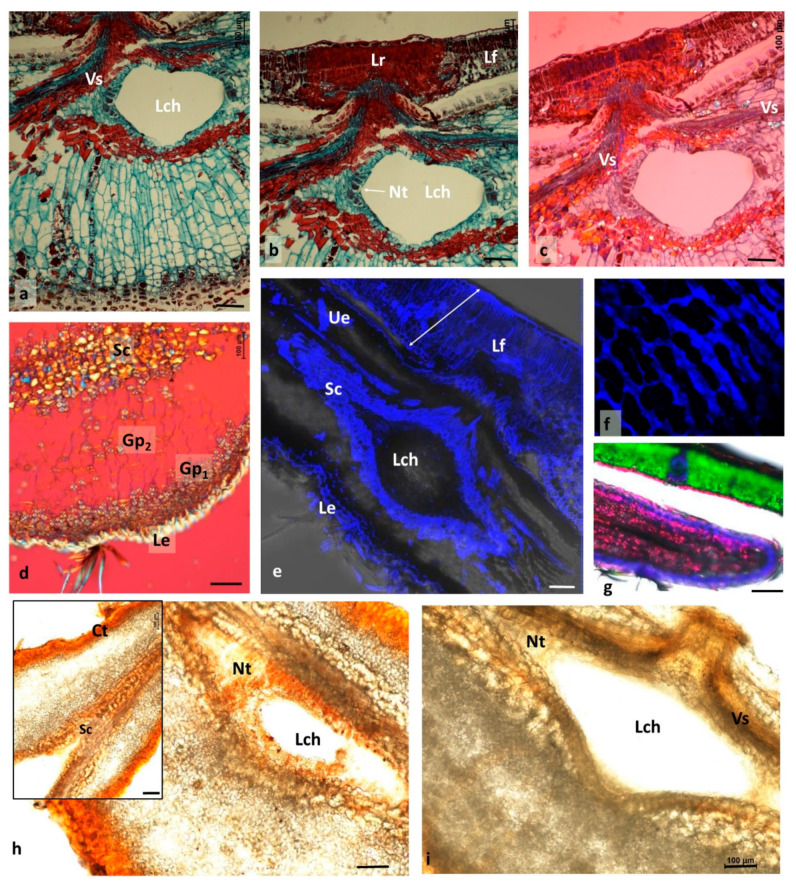
Autumn spangle gall (LM and FM—polarized light; a–c fast green and safranine). (**a**) Cross-section through the centre of the mound. Dark red, lignified parts; Vs, vascular strands; Lch, larval chamber. (**b**,**c**) Cross-section through the place of coalescence (Lr) between gall tissue and leaf; Nt, nutritive tissue; (**b**) normal light; (**c**) polarized light. (**d**) Cross-section through the mound of the gall. Starch accumulated near the epidermis (Gp_1_); in the centre, it rarely occurred (Gp_2_). (**e**) Cross-section through gall and sustaining leaf (FM, blue—lignin autofluorescence). Arrow with two heads, leaf tissue; Ue, adaxial epidermis; Le, abaxial epidermis; Sc, sclerenchyma. (**f**) Abaxial epidermis (FM), mainly anticlinal walls, lignified (blue). (**g**) Marginal part of a gall and sustaining leaf in cross-section. (FM) Green, strong green autofluorescence of leaf chlorophyll; blue, lignin autofluorescence; pink, tannin autofluorescence. (**h**) Cross-section through larval chamber (stained with Sudan III); orange-red coloration, lipids (including cutin and waxes). Insert, another place in the same gall; (**i**) cross-section through larval chamber stained with KNO3—yellow–grey, the proteins. Bars represent 100 µm.

The larval chamber contained nutritive tissue (Figure 4e,f). In the relatively young galls at the end of July, cell walls were not observed (Figure 4e,f). The nutritive tissue sometimes had a granular structure (Figure 4e). The cross-section presented in Figure 4g was cut when the cellular structure in nutritive tissue started to be visible. In a gall collected one week later (Figure 4i), the nutritive tissue showed an already fully developed structure. Its cells had been vertically extended between the two plates of sclerenchyma. In the autumn galls (Figure 5a), the nutritive tissue formed a compact tissue that filled the corners between the two sclerenchymatous plates; besides that, a thin layer of nutritive tissue not yet consumed by the larva lined the larval chamber. The larval chamber has almost attained its definitive size at that time.

Analysing the arrangement of parenchymatous cells in the cross-section through the mound (Figure 5a) showed that cells were organized in parallel rows starting from the sclerenchyma (surrounding the larval chamber) and terminating in the subepidermal layers of cells. These subepidermal cells contained dense cytoplasm and a distinct nucleus, and pairs of cells that appeared to be formed as a result of recent divisions.

Another trait of the mound parenchyma was the very scarce occurrence of starch in its central part (Figure 5d). Starch occurred in larger amounts only in the parenchyma of the subepidermal region close to the cells, showing meristematic characteristics, and sometimes also near the sclerenchyma surrounding the larval chamber.

The gall strongly influenced the tissue of the subtending leaf. Its influence was initially only visible at a distance of 1 to a few millimetres around the gall stalk but expanded with time. We now present the situation from the middle of August to early September. The place of coalescence of the gall with the leaf in normal and polarized light is shown in Figure 5b,c. Gall tissue penetrated the leaf in the form of a cone. Leaf tissue around this cone was markedly changed. The spongy parenchyma was much thicker than normal, and the tissue was very compact, which indicates that many additional cell divisions had occurred (hyperplasia). Staining with safranine and fast green, and the application of polarized light revealed strong lignification of the spongy parenchyma. Palisade parenchyma was also changed: cells were very compact, which means that many additional anticlinal cell divisions had occurred in that tissue (Figure 5b,c). Lignification of the walls in the palisade parenchyma was also conspicuous. In some cases, the influence of the gall on the leaf was very strong: leaf cells were deformed and strongly stained with safranine (Figure 4b,c). The normal leaf histology was apparent only at some distance from the gall (Figure 4h).

Localization of lignified elements in an autumn gall and in its sustaining leaf is shown in Figure 5e. Adaxial cell walls were lignified, especially the abaxial epidermis and the inner leaf cells (palisade and spongy parenchyma) situated near the gall stalk. Strongly lignified was also the sclerenchymatous lenticular capsule in cross-section surrounding the larval chamber. In the abaxial epidermis of the gall (Figure 5f), mainly the anticlinal walls were lignified. Strong green chlorophyll autofluorescence in an oak leaf and pink tannin autofluorescence in the marginal part of a gall are shown in Figure 5g.

In order to know more about the biochemical nature of the nutritive tissue of the gall, we applied Sudan III to the central cross-section of a gall (Figure 5h). An orange-red coloration was an indication that nutritive tissue contained fats. Epidermal and subepidermal layers showed strong orange-red coloration due to the presence of cutin. In the HNO3 test for proteins (Figure 5i), the nutritive tissue stained yellow–grey, which means that it contained reserve proteins. Yellow coloration also suggests a higher content of proteins in the epidermis and in cells contiguous to the vascular strand (Vs). The sclerenchyma above and below the larval chamber was pale yellow.

### 3.4. Older Galls Collected in Autumn—TEM Observations

**Figure 6:**
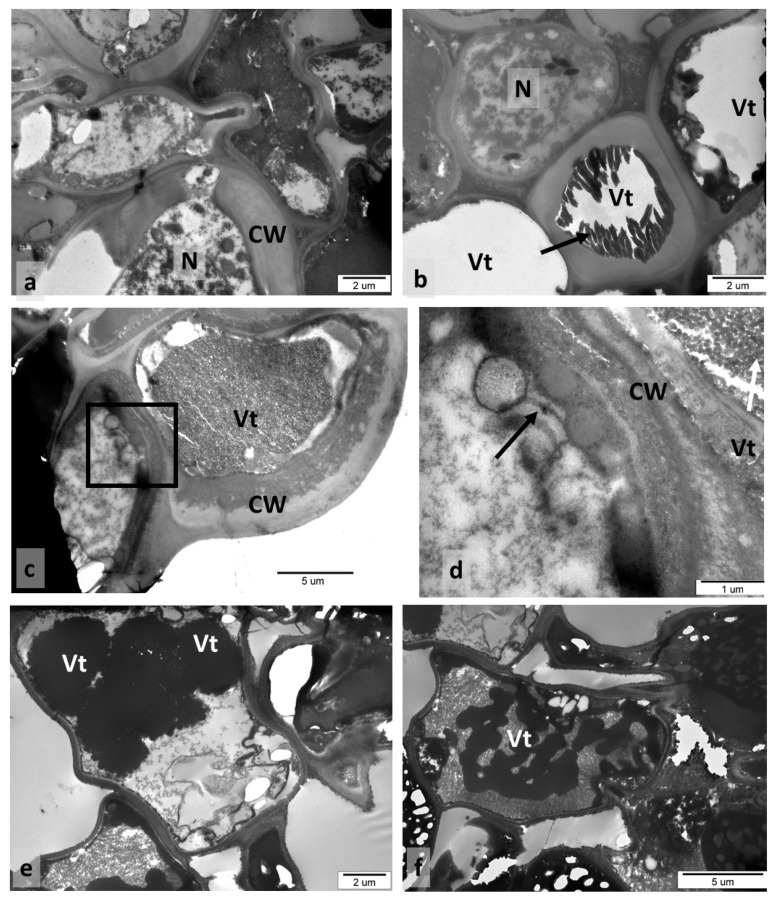
Autumn galls (TEM images). (**a**) Cross-section taken at border between sclerenchyma and gall parenchyma (CW, cell wall; N, nucleus). (**b**) Vt, cell tannin content of vacuoles was damaged during preparation. (**c**) Two cells of abaxial epidermis. Tannin accretions were observed in the vacuole of the larger cell and in its external cell wall. In the second cell, there was a partly visible heavy deposit of tannins in the cell wall. (**d**) Detail of previous picture. White arrow, granular structure of tannin accretions; black arrow, vesicle-like structures. (**e**) In this cell, dense tannin deposits were in each of the five vacuoles, other vacuoles without tannins. (**f**) Tannin deposits in the form of a band.

The cross-section presented in Figure 6a was taken at the border between the sclerenchymal capsule and the gall parenchyma, so cells with very thick walls were found side by side with thin-walled ones. Both parenchymal and sclerenchymal cells were alive, and the latter contained large nuclei and a dense cytoplasm (Figure 6a). Some cells had tannin accretions as strong osmiophilic precipitations, especially localised in tannin vacuoles (Figure 6a–e). A single large tannin vacuole (Figure 6b,c) or few small vacuoles with tannins (Figure 6e) were visible in the gall cells. Different tannin forms were found: with a granular structure filling the whole vacuole (Figure 6c,d), as uniform and very electron-dense (Figure 6e), or in the form of dark streaks (Figure 6f). Tannin was also found in the cell walls (Figure 6c,d). In addition to tannin vacuoles, vacuoles without tannins, electron-transparent content, were also present in the gall cells.

## 4. Discussion

Structural studies are very important for understanding insect–plant interactions, since structure is closely related to function. The spangle gall (*N. quercusbaccarum*) was described by Hough [27] (see Meyer and Maresquelle [3]; Kovácsné Koncz et al. [30]); however, knowledge of their structure is insufficient. In our paper, we presented the description of spangle-gall anatomy in late July in the stage corresponding to Stage 11 of Hough’s text [27], and in the middle of August and autumn (September).

The outer surface of a gall that was exposed to the influence of the environment was covered with tufted (stellate) hairs protruding in all directions. They were lignified and might present an obstacle for potential predators. Tufted hairs are the characteristic trait of the spangle gall. They are similar to hairs found on *N. numismalis* galls. The main difference is that, in spangle galls, the hairs occur in clusters; in *N. numismalis* galls, they form a wide ring around the “bald” central plane [23]. As in *N. numismalis*, the spangle-gall hairs were transformed epidermal cells [27].

We found two types of openings on the outer surface of the gall. The first, sporadically observed in young galls, was stomata-like. For comparison, in a typical oak leaf, stomata are very densely located on its abaxial side [23]. Similarly, small openings were also observed on the abaxial side of *N. numismalis* galls [23]. They are unlikely to be related to aeration of internal gall tissue. Because they are rare and small, one would expect hypoxia inside the young gall. The openings of the second type were relatively large and situated on top of multicellular epidermal protuberances. Similar openings were also found on *N. numismalis* galls [23]; however, their aperture was much narrower, and epidermal protuberances were much smaller. Large openings on epidermal protuberance resemble to some extent “stomata at top of a column of cells” [40] observed, for example, on the overgrown sepals of *Physalis ixocarpa* Brot. [41]. To our knowledge, this is the first report of the presence of large epidermal protuberances with wide openings on the surface of a mature gall. Such large openings may markedly facilitate the aeration of internal gall tissue. On the other hand, their formation is associated with the breaking of epidermal integrity, which increases the risk of infection by microorganisms, creating a gate for invasion.

In spangle galls, like in other cynipid galls, tissue formed concentric layers around the larval chamber [42]. The location of cells with CaOx crystals seemed unique in the galls. Such cells were only visible around the plates of the sclerenchyma capsule encasing the larval chamber from above and below. The occurrence of CaOx crystals is common in plants, and they are observed in the tissue of almost all plants [43,44,45]. CaOx crystals have many functions in plants, including bulk calcium regulation, the regulation of ion balance (e.g., sodium and potassium balance), protection against phytophagous organisms, plant-tissue rigidity, detoxication of heavy-metals and of oxalic acid. They also show light-gathering and reflection effects [43,44,45,46]. CaOx crystals may also act as an internal reserve of carbon that is used in photosynthesis when sufficient atmospheric carbon dioxide is not available, functioning as a dynamic carbon pool [47]. In spangle galls, cells containing crystals of calcium oxalate (CaOx) may strengthen the function of the sclerenchymal stratum around a larval chamber. They could play a role as a kind of barrier against the invasion of parasites, plant consumers, or inquilines. CaOx crystals have detrimental effects on chewing insects, but not on sap-feeding insects with piercing–sucking mouthparts [48]. In spangle galls, larvae are protected in many ways. A complex barrier system was created by numerous clusters of hairs on the gall surface, lignified epidermal cells with a thick cuticle, and two plates of sclerenchymal capsule with CaOx crystals encasing the larval chamber from above and below.

Spangle galls grow during the vegetation phase due to the activity of the gall marginal meristem [3,27]. The arrangement of epidermal gall cells in centrifugal rows (dividing dichotomically from time to time) fully confirms this statement (Figure 2c). As the gall disk grows, new cells are added at the end of each row of cells. Moreover, cell divisions also occur in the body of the gall because disk thickness increases as the gall grows (Figure 4a,b). According to Hough [27], periclinal divisions also occur in subepidermal layers, adding new cells that increase the thickness of the gall. However, the arrangement of cells in the mound of the gall (Figure 5a) indicates that there is an additional meristematic area in the abaxial subepidermal tissue of the gall mound. The intensive cell divisions inside it produce long vertical rows of cells stretching up to sclerenchyma.

The spangle gall also has a period of active development during the cold part of the year. An insect enclosed in a gall spends this time under the cover of fallen leaves. For this period, sufficient food reserves must be accumulated. Some food reserves are stored in the gall parenchyma in the form of starch (Figure 2g), and in the form of proteins and lipids in the nutritive tissue (Figure 5h,i).

In the nutritive tissue, we observed an unexpected phenomenon: when the gall was young, its larval chamber contained a granular substance without visible traces of cellular structure (Figure 4e,f). The distinct cellular structure appeared only during later development (Figure 4g,i). The cells that appeared were vertically extended between the upper and lower sclerenchymatous plates. During autumn, the nutritive tissue formed compact groups of cells in the corners of the larval chamber, or it lined the inside of the larval chamber (Figure 5a–c). The analogy in plants to the noncellular structure of the tissue containing reserves of nutrients is the endosperm in seeds [49]. Hough [27] presented the cellular structure of the nutritive tissue, so if we used some special staining methods, the cell walls could be visible. However, in some early stages of nutritive-tissue development, the cellular walls dissolve [14]. Bronner [14] described the dissolution of whole cells below the eggs of *N. numismalis* (see Meyer and Maresquelle [3]). The cavity in leaf tissue appears there, and the larvae enter it. So, it should be not surprising that young larvae can cause the dissolution of cell walls in the nutritive tissue. Taking into consideration that gall-making larvae are initially very small, and their mouth apparatus is delicate, the dissolution of the cell walls in nutritive tissue would be advantageous for it.

Histochemical analyses confirmed earlier statements that the nutritive tissue of galls was rich in proteins and lipids. We did not detect starch grains in the nutritive tissue, so proteins and fats may be the key products for larval nutrition during the early stages of development. We detected abundant starch reserves in parenchymal cells that could serve as a reserve for larvae and gall tissue, especially during overwintering.

The abundance of tannins is characteristic of oak leaves and different oak galls [2,3]. In epidermal gall cells, tannins occur in a granular form. This concurs with the results of Brillouet et al. [50], who showed that tannin accretions are formed of small tannin “shuttles”. These authors reported that, in tracheophyta, tannin accretions “must no longer be viewed as reservoirs integrally and homogenously filled with tannins forming a continuous phase”, but rather as granular structures. According to Fleurat-Lessard et al. [51], and He et al. [46], tannins are mainly present in vacuoles, but are also found in cell walls, for instance, in grape berry skin and in seeds. Our results confirmed this view. Tannins detected in *Quercus* leaves were localised in chloroplasts, cell walls, and intercellular spaces [46]. In spangle galls, the tannins commonly occurred in vacuoles and cell walls. Concerning the possible role of condensed tannins in plant defence, Barbehenn and Constabel [52] stated that tannins have little association for defence against populations of the large majority of oak-feeding insects. However, the inhibitory effects of tannins on pathogenic bacterial growth were demonstrated by González-Lamothe et al. [53]. The antimicrobial action of tannins was discussed by Scalbert [54]. Recently, tannins and their degradation product, gallic acid, attracted attention as raw material in pharmaceutical and other industries [55]. Tannins are also assessed as natural products that, thanks to their antimicrobial properties, may replace infeed antibiotics [56].

The structure and physiology of galls changes as they grow and develop. They are synchronized with the development of larvae. The fundamental function of a gall is to feed and defend larvae; as our observations showed, it was associated with cell and tissue modification to ensure optimal living conditions. The changes included strengthening the protective barrier, adjusting the quality and availability of the food base (cell lysis) for larvae, or modifying the epidermis to facilitate gas exchange. The coordination of complex developmental processes of larvae and their galls requires a continuous molecular dialogue between them.

## 5. Conclusions

Differences between the structure of the spangle gall and the oak leaf were shown. The gall contains highly lignified tufted hairs, the epidermis is almost deprived of normal stomata, and the large openings on the top of epidermic protuberances were observed that had not been described in the literature. A sclerenchymatous capsule with many CaOx crystals that protect the larval chamber was also observed. We found an additional meristematic area in the abaxial subepidermal tissue of the gall mound, active long after the leaf cells had terminated their divisions. Changes in the structure of nutritive tissue were observed, starting from the granular matrix in which cell walls were not visible, through the tissue with very distinct vertically elongated cells, up to the “compact tissue” in the corners of the larval chamber in a mature gall. This study adds to the knowledge on anatomical and ultrastructural changes during spangle-gall development; however, the detailed mechanism underlying spangle-gall induction by *N. quercusbaccarum* requires further study.

## Data Availability

The data presented in this study are available in article.
